# TGF-*β* Signaling Pathway-Based Model to Predict the Subtype and Prognosis of Head and Neck Squamous Cell Carcinoma

**DOI:** 10.3389/fgene.2022.862860

**Published:** 2022-05-02

**Authors:** Lian Zheng, Zhenjie Guan, Miaomiao Xue

**Affiliations:** ^1^ Department of Oral and Maxillofacial Surgery, The First Affiliated Hospital of Zhengzhou University, Zhengzhou, China; ^2^ Department of Stomatology, The First Affiliated Hospital of Zhengzhou University, Zhengzhou, China; ^3^ Department of General Dentistry, The First Affiliated Hospital of Zhengzhou University, Zhengzhou, China

**Keywords:** TGF-*β*, head and neck squamous cell carcinoma, subtype, gene model, prognosis

## Abstract

**Background:** Although immunotherapy with immune checkpoint therapy has been used to treat head and neck squamous cell carcinoma (HNSCC), response rates and treatment sensitivity remain limited. Recent studies have indicated that transforming growth factor-β (TGF-β) may be an important target for novel cancer immunotherapies.

**Materials and methods:** We collected genomic profile data from The Cancer Genome Atlas and Gene Expression Omnibus. The least absolute shrinkage and selection operator method and Cox regression were used to establish a prognostic model. Gene set enrichment analysis was applied to explore biological functions. Tracking of indels by decomposition and subclass mapping algorithms were adopted to evaluate immunotherapy efficiency.

**Result:** We established a seven TGF-β pathway-associated gene signature with good prediction efficiency. The high-risk score subgroup mainly showed enrichment in tumor-associated signaling such as hypoxia and epithelial-mesenchymal transition (EMT) pathways; This subgroup was also associated with tumor progression. The low-risk score subgroup was more sensitive to immunotherapy and the high-risk score subgroup to cisplatin, erlotinib, paclitaxel, and crizotinib.

**Conclusion:** The TGF-*β* pathway signature gene model provides a novel perspective for evaluating effectiveness pre-immunotherapy and may guide further studies of precision immuno-oncology.

## Introduction

Head and neck squamous cell carcinoma (HNSCC), originating from the oral cavity, oropharynx, larynx, and hypopharynx and displaying rapid progression, has become a significant human health problem (Siegel et al., 2020; Wang Z. et al., 2021; Jia et al., 2021). More than 600,000 new cases of HNSCC are diagnosed worldwide annually (Siegel et al., 2020). With high malignancy, rapid progression, and poor prognosis, HNSCC has become the sixth most common cancer worldwide. The first choice for HNSCC treatment is still surgical salvage, followed by postoperative chemo-and/or radiotherapy (Shibata et al., 2021). The recurrence rate following HNSCC treatment is high at 25–50%, depending on the location of the tumor, the clinical stage and grade and HPV infection status (Ho et al., 2014). Treatments of patients with locally advanced HNSCC remains great challenge (Ang et al., 2014). In recent years, some patients with cancer have benefited from immunotherapy (Naidoo et al., 2021). Indeed, immunotherapies have been approved and widely used for recurrent and metastatic HNSCC; however, only a relatively small subset of patients, approximately 15–20%, truly benefit from this approach (Lee et al., 2020). Therefore, exploring the immune microenvironment and immune resistance mechanisms is crucial and provides support for evidence-based treatment decisions. Overall, exploring the genome and microenvironment of HNSCC might provide clues for identifying biomarkers predicting the effectiveness of immunotherapy. microenvironment. studies have demonstrated that esophageal adenocarcinoma cells and xenograft tumors can be resistant to trastuzumab and pertuzumab by activating TGF-β signaling, which induces epithelial-mesenchymal transition. Thus, block TGF-β signaling can increase the anti-tumor efficacies of trastuzumab and pertuzumab in esophageal adenocarcinoma cells and xenograft tumors (Ebbing et al., 2017; Ebbing et al., 2019; Steins et al., 2019). Thus, targeting the TGF-β pathways may benefit from chemical resistance. As TGF-β pathway-associated genes are important in the response to tumor therapies, modulating TGF-β-associated pathway activities and expression of related genes may greatly impact tumor malignant abilities. TGF-β comprises a family of growth factors, which play crucial roles in development, fibrosis, and cancer progression (Nüchel et al., 2018). TGF-β binding activates type II and then type I receptors, that in turn activate an increase of SMAD signals activation (Petiti et al., 2018; Lähde et al., 2021). Studies have demonstrated that high cancer-associated fibroblast infiltrated gastric cancer is associated with immunosuppressive microenvironment regarding to TGF-β alterations (Liu et al., 2021). TGF-β is also involved in tumor metabolic and immune microenvironment. The TGF-β inhibition can also promotes tumor cell death thus obtaining an effective anticancer immunotherapy immune response (Huang et al., 2021). TGF-β family genes are crucial immune suppression genes in head and neck cancer. These genes were associated with decreased survival probability of head and neck cancer (Budhwani et al., 2021). This suggesting that TGF-β associated pathway have potential become an attractive target for future cancer therapy.

In this study, we comprehensively examined TGF-β-associated genes and related immune infiltration in HNSCC, evaluating their clinical significance in predicting prognosis and evaluating therapies effectiveness.

## Materials and Methods

### Data Collection

**FIGURE 1:**
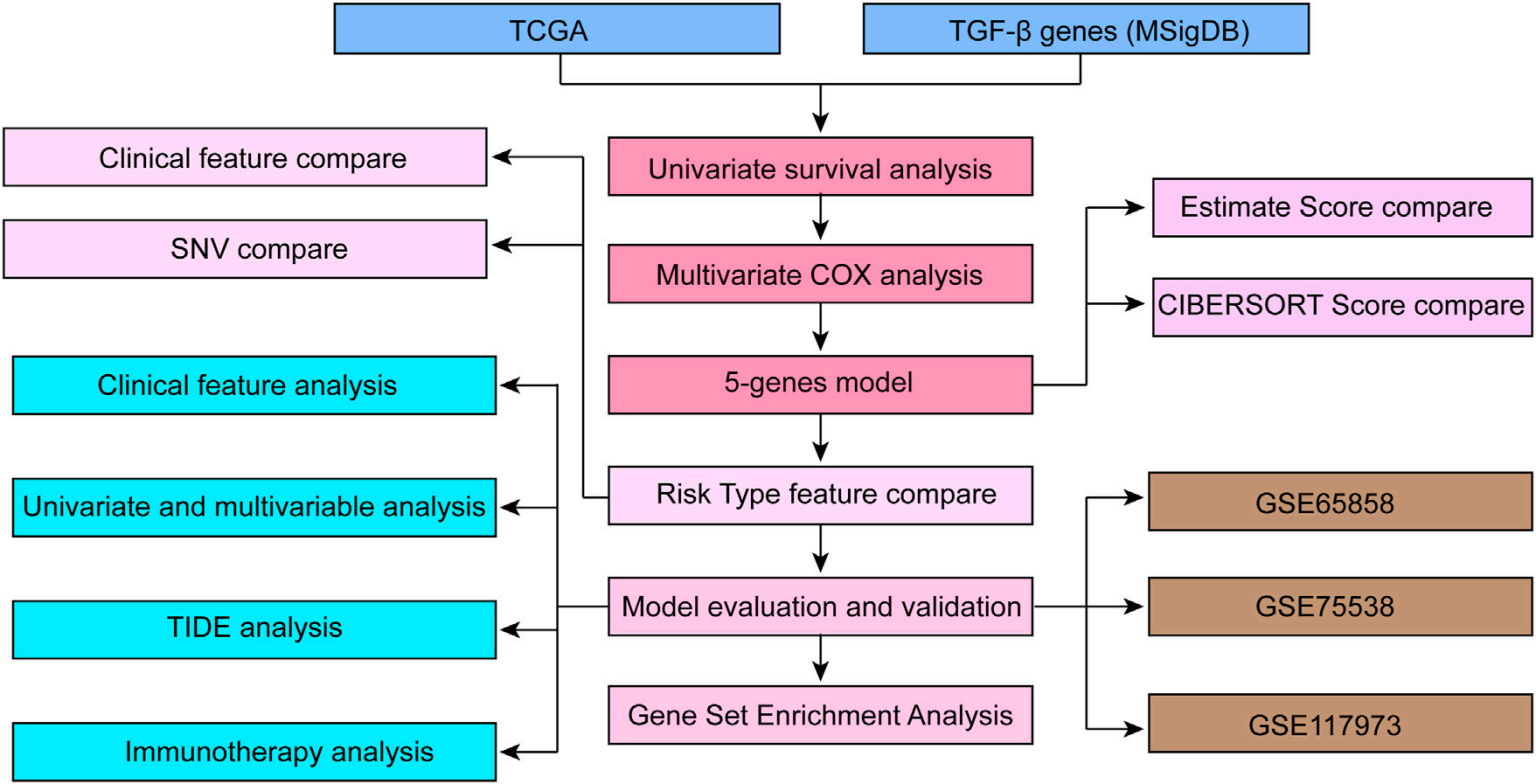
The workflow of TGF-β pathway signature-associated HNSCC prognosis analysis.

Flowchart of the study protocol of TGF-β-related characteristics related to the prognosis of HNSCC is listed in Figure 1. We used open datasets from The Cancer Genome Atlas (TCGA) (https://cancergenome.nih.gov/) and Gene Expression Omnibus (GEO) (https://www.ncbi.nlm.nih.gov/geo/) databases, including the GSE65858, GSE75538 and GSE117973 chip datasets. The TGF-β mRNA expression status and correlating tumor immune microenvironment evaluation indicators in HNSCC and corresponding normal tissues were analyzed through the SangerBox database (http://sangerbox.com/Tool). We identified the *p* value as 0.001, with a fold change of 1.5.

### Data Preprocessing

RNA-seq data from TCGA HNSCC and GEO HNSCC with unreliable and incomplete clinical data were removed. The clinical statistical information of HNSCC patients after data pretreatment is shown in Table 1.

**TABLE 1:** The clinical information of HNSCC patients from different cohorts.

Clinical features	TCGA-HNSC	GSE65858	GSE75538	GSE117973
OS/PFS				
Alive	297	176	11	55(PFS)
Dead	220	94	3	22(PFS)
Gender				
Female	136	47	6	17
Male	381	223	8	60
T stage				
T1	36	35	-	7
T2	149	80	-	30
T3	136	58	-	17
T4	184	97	-	23
N stage				
N0	244	94	-	36
N1	83	32	-	17
N2	162	132	-	24
N3	9	12	-	0
M stage				
M0	491	-	-	-
M1	6	-	-	-
Stage				
I	27	18	-	-
II	81	37	-	-
III	93	37	-	-
IV	316	178	-	-
Age				
≤65	340	184	12	53
>65	177	86	2	24
Grade				
G1	61	-	-	1
G2	303	-	-	46
G3	124	-	-	30
G4	7	-	-	0

### Prognostic Risk Model Construction Based on TGF-β Pathway Genes

To construct the TGF-β associated prognostic model, we downloaded HNSCC transcriptional data from public database. All selected data were screened and selected with complete gene expression information and clinical information. We applied 54 TGF-β -related genes, univariate survival analysis and Lasso Cox multivariate analysis to determine the risk score of seven key genes. High and low risk groups are divided by selecting the best threshold. Therefore, we determined that λ = 0.0086 would obtain the optimal model. In addition, we selected seven genes at λ = 0.0086 as targets for the next step. The final 7-gene signature formula is as follows: risk score = 0.0003 ∗ BCAR3-0.062 ∗ ID2 + 0.112 * NOG +0.009∗ SERPINE1 + 0.177 SLC20A1 + 0.106 ∗ THBS1-0.351 ∗ TR IM33.

The HNSCC RNA-seq data from TCGA were identified as the training set. We adopted univariate Cox proportional risk regression to construct and predict a TGF-β-associated gene model (54 in total). Overall survival data were analyzed by using the R package survival COXPH function. We defined the threshold for filtering as *p* < 0.05. In this study, we selected five genes with hazard ratios (HRs) larger than one and two genes with HRs less than 1.

### Univariate and Multivariate Cox Regression Analyses

Univariate Cox regression analyses of the correlation between TGF-β-associated gene expression and HNSCC clinical prognosis information were carried out by Cox proportional hazards regression analysis. Genes with *p* < 0.05 were considered significant. Determination of genes in the TGF-β-specific module closely related to prognosis in HNSCC were explored.

### Cox Regression Analysis

We utilized the R software package glmnet for least absolute shrinkage and selection operator (LASSO) Cox regression analysis to determine the greatest impact on the prognosis of HNSCC. LASSO applies an L1-regularization penalty, ρ, to estimate a penalized precision matrix to illustrate indicators with the highest contribution (Love et al., 2016). In the LASSO model, the minimum criterion (λ) based on 10-fold cross validations and 1,000 iterations is chosen. The selected genes were then included in a multivariate Cox regression model, and those gene sets with the best prognostic value were identified by positive selection and reverse elimination methods.

### Prognosis Prediction of TGF-β-Associated Genes

To determine correlations between TGF-β-associated gene expression and HNSCC prognosis, we applied the Wilcoxon test and divided HNSCC patients into two subgroups based on TGF-β expression level. The optimal cutoff point for gene expression was obtained based on the R package “survminer” (cutoff = −0.007). The threshold is −0.007, in which the groups with a risk score greater than −0.007 were high risk groups, and those with a risk score less than −0.007 was identified as low risk groups We used Kaplan-Meier curves to evaluate the prognostic value of various clinical features through the R package ‘survminer’ (CRAN.R-project.org/package = survminer). This method has been described in a previous study (Alcala et al., 2019; Zhuang et al., 2020).

### Somatic Mutation Analysis

To evaluate somatic mutations in HNSCC, we applied the package TCGA biolinks in R and downloaded mutation annotation files. Somatic single-nucleotide polymorphisms and indels in tumor samples were called using the MuTect2 (http://www.broadinstitute.org/cancer/cga/mutect) pipeline Genome Analysis Toolkit (GATK; Broad Institute, Cambridge, MA, United States). We counted differences in the number of mutant genes in the samples. Furthermore, we screened out genes with mutation frequencies greater than three and used the chi-square test to screen genes with significantly high-frequency mutations in each subtype, with a selection threshold of *p* < 0.05.

### Gene Set Enrichment Analysis (GSEA)

GSEA was performed to examine different biological processes with GSEA software (http://software.broadinstitute.org/gsea). Gene Ontology (GO) and Kyoto Encyclopedia of Genes and Genomes (KEGG) were used for hierarchical analysis for high- and low-risk groups of HNSCC patients. The R package clusterProfiler (https://guangchuangyu.github.io/software/clusterProfiler) (v3.14.0) was employed to process the KEGG and GO analyses. We identified the number of random sample permutations as 1,000, and enriched gene sets with a nominal *p* < 0.05 and 25% cutoff on false discovery rate (FDR) were defined as significant.

### Immune Infiltration Scores Estimation

We applied the “ESTIMATE” R package to assess overall immune infiltration indicators based on the medium scores of ImmuneScore, StromalScore, and ESTIMATEScore (Ghatalia et al., 2019). We also calculated the proliferation score of all cells. These proliferation scores were obtained from previous study (Thorsson et al., 2018).

### Tumor Immune Dysfunction and Exclusion (TIDE)

The TIDE algorithm was used to link individual immunotherapy responses with the TIDE web tool (Netherlands Cancer Institute, Amsterdam, Netherlands, available from http://shinyapps.datacurators.nl/tide/). TIDE is used to estimate the spectrum and frequency of small insertions and deletions (indels) generated in a pool of cells by genome editing tools such as CRISPR/Cas9, TALENs and ZFNs. In this study, we applied TIDE to estimate the likelihood of immunotherapy response.

### Cell Culture and Quantitative Real-Time Reverse Transcriptase–Polymerase Chain Reaction

The human nasopharyngeal carcinoma cell NPC and human immortalized nasopharyngeal epithelial cell NP69 were purchased from FuHeng (Shanghai, China). The cell line NP69 was cultured in KM medium, and the cell line NPC was cultured in Dulbecco’s modified Eagle medium supplemented with 10% fetal bovine serum (ThermoFisher Scientific, Waltham, MA, United States). Cell lines were grown at 37 °C in a humidified incubator containing 5% CO_2_.

Total RNA was extracted from NP69 and NPC cells using TRIzol reagent (Invitrogen Life Technologies, Waltham MA, United States), followed by reverse transcription according to the manufacturer’s instructions (Takara, Japan). The specific quantitative primers used are listed in Supplementary Table S1. Samples were assessed by quantitative real-time reverse transcriptase–polymerase chain reaction (qRT-PCR) using an Agilent Mx3005P using SYBR qPCR Mix (MQ10201s, Monad Biotech, Wuhan, China). Human glyceraldehyde 3-phosphate dehydrogenase (*GAPDH*) was used as an endogenous control. Relative expression levels were defined according to the 2^−ΔΔCt^ method. Each experiment was performed in triplicate.

### Statistical Analysis

We calculated correlations with the Pearson correlation coefficient, and differences between subgroups were determined by the Wilcoxon test or Kruskal–Wallis test. The data are expressed as means ± SD. A two-tailed *p*-value less than 0.05 was defined as statistically significant.

## Results

### Univariate and Multivariate Risk Analyses of the Training Set

The workflow of this study is depicted in Figure 1. To select proper protective factors and risk factors, we applied TCGA-HNSCC datasets as a training set; GSE65858, GSE75538, and GSE102995 were used as external independent validation datasets. These datasets were obtained from GEO database, which were used for verified the accuracy of the model.

### Risk Model Construction and Validation

**FIGURE 2:**
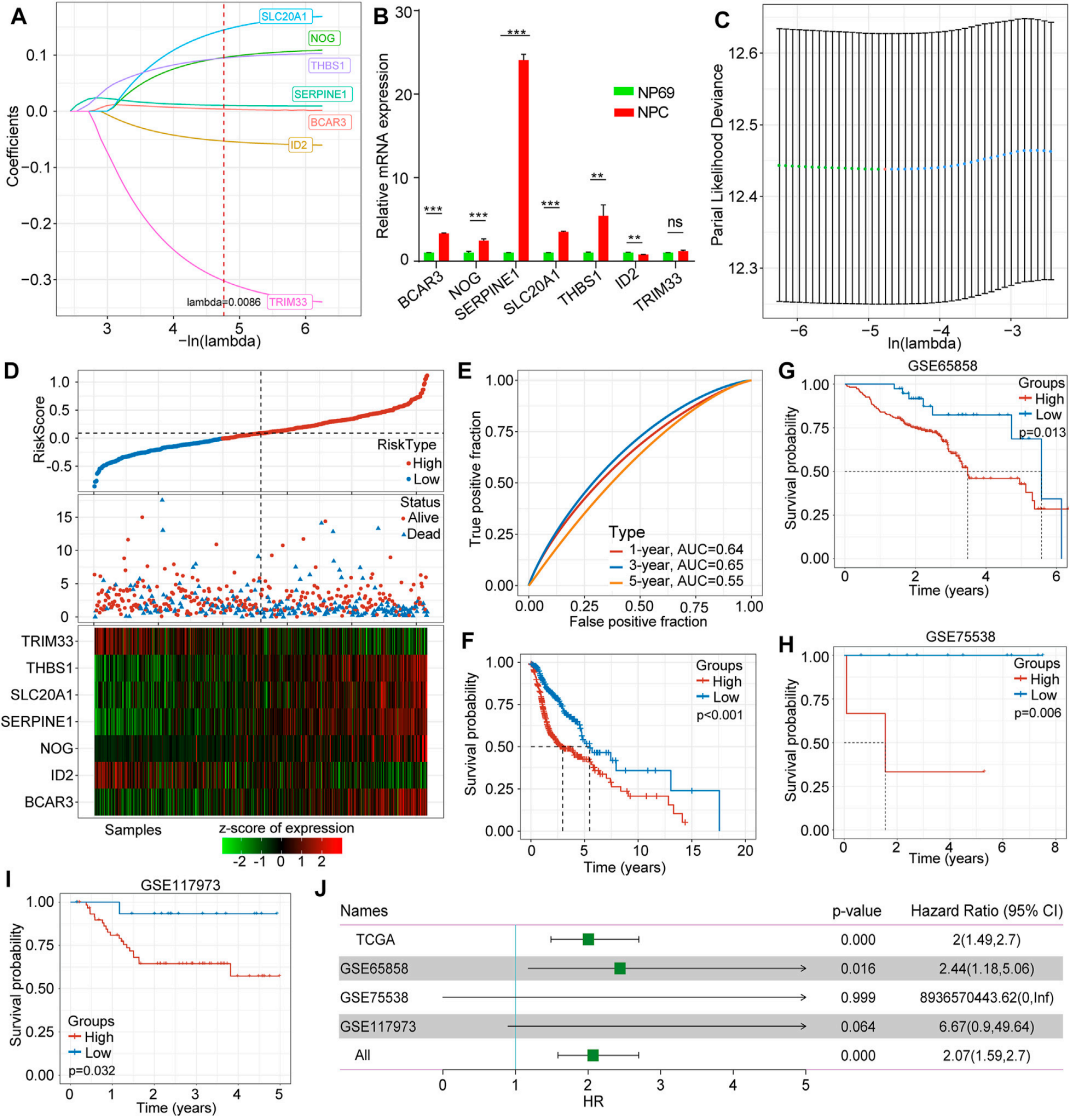
Construction and validation of the TGF-β-associated prognostic risk score model. **(A)** The trajectory of each selected gene in TGF-β pathways. **(B)** Expression levels of hub genes in human nasopharyngeal carcinoma cell NPC and human immortalized nasopharyngeal epithelial cell NP69 by qRT-PCR. **(C)** The confidence interval of each lambda. **(D)** Correlations and distribution of risk score, survival time and survival status and expression levels of the seven selected genes. **(E)** The classification efficiency of prognostic prediction for HNSCC at 1 year, 3 years, and 5 years. **(F)** The survival curve between high- and low-risk scores of the seven-gene signature. **(G)** The survival curve between high- and low-risk score subgroups in GSE65858. **(H)** The survival curve between high- and low-risk score subgroups in GSE755538. **(I)** The survival curve between high- and low-risk score subgroups in GSE117973. **(J)** Forest plots of risk scores in different datasets.

**FIGURE 3:**
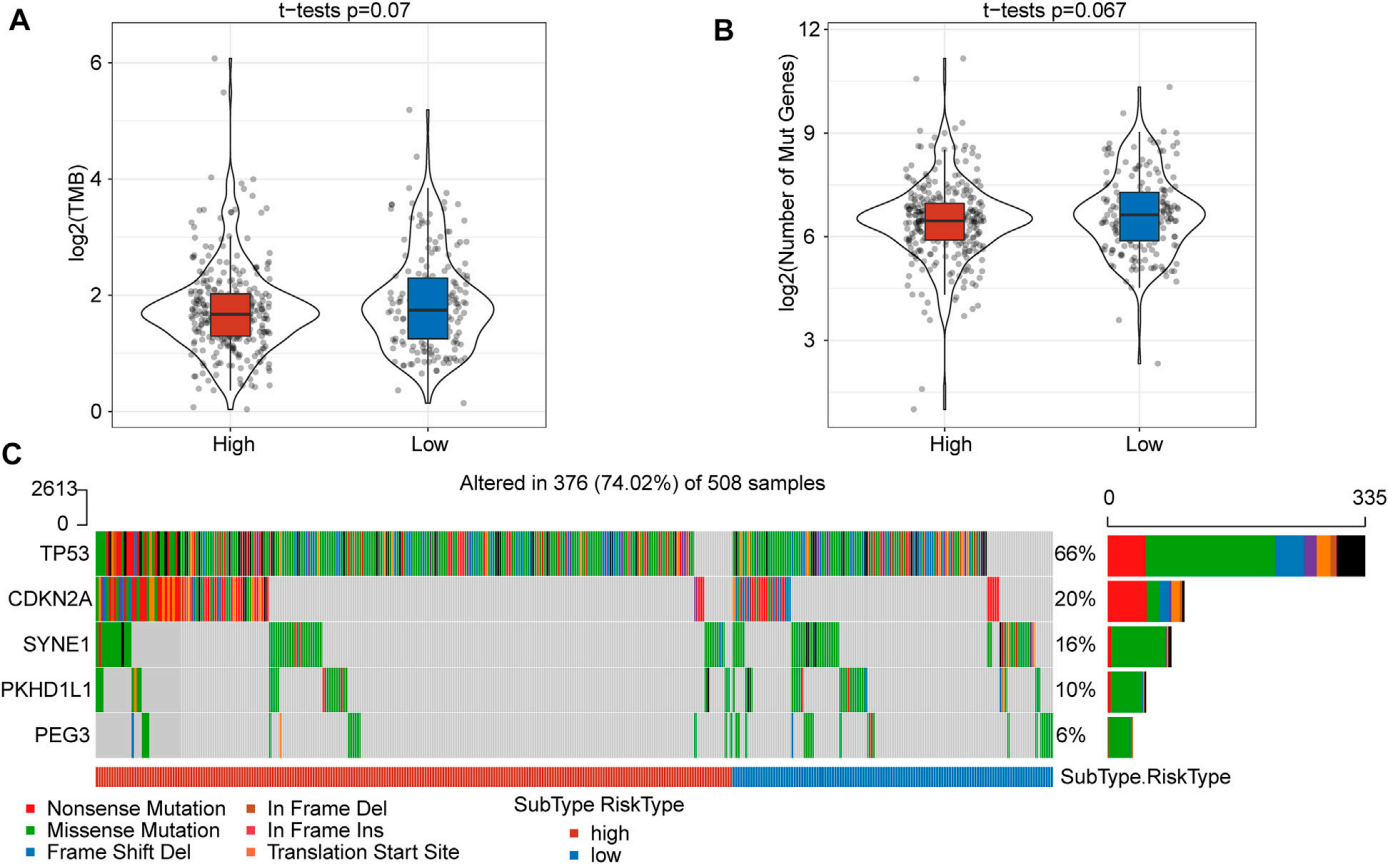
The TMB and somatic mutation information between high- and low-risk score subgroups. **(A)** TMB score between the high- and low-risk subgroups showed no significant difference. **(B)** The number of mutated genes between the high- and low-risk subgroups was not significantly different. **(C)** Mutation characteristics of significantly mutated genes in the subgroups.

We adopted LASSO Cox regression to obtain the change trajectory of each independent variable, as illustrated in Figure 2A. The expression levels of the identified seven genes in human nasopharyngeal carcinoma cell NPC and human immortalized nasopharyngeal epithelial cell NP69 are shown in Figure 2B. We applied 10-fold cross-validation techniques to avoid performance bias with all prediction methods (Figure 2C). We also calculated the risk score for each sample in the dataset TCGA-HNSCC according to the TGF-β expression level and plotted the risk score distribution of the samples, as shown in Figure 2D. The different expression of seven different signature genes with the increase of risk value was assessed. High expression of BCAR3, NOG, SERPINE1, SLC20A1, and THBS1 was identified as a risk factor associated with a high risk score. Conversely, high expression of ID2 and TRIM33 was associated with low risk, constituting a protective factor (Figure 2D). Furthermore, we used the R software package and receiver operating characteristic (ROC) curve analysis to evaluate prognostic factors. We analyzed the classification efficiency for prognosis prediction, and areas under the curve (AUCs) at 1, 3 and 5 years were 0.64, 0.65, and 0.55, respectively, as indicated in Figure 3C. An AUC = 0.64 at 1 year was the most reliable indicator for survival prediction with this 7-gene model (Figure 2E). To further investigate clinical prognosis between the high- and low-risk score subgroups, we applied the R package “survminer” with the cutoff = -0.007. The Kaplan-Meier curve indicated that the difference between the high-and low-risk score subgroups was highly significant (*p* < 0.0001) (Figure 2F). In total, 318 samples were assigned to the high-risk group and 199 to the low-risk group. We downloaded validation datasets such as GSE65858, GSE75538, and GSE102995 and applied the same gene models and coefficients. As the GSE65858 dataset lacked NOG gene expression, we used a six-gene model in further validation. The results of the three external validation datasets were highly consistent with those of the training sets. Overall, the high-risk score subgroup had a poorer prognosis than the low-risk score subgroup (Figures 2G–I). The forest plot showed that TGF-β signaling pathway-associated gene-based characteristics might be major risk factors for HNSCC (HR = 2.069, 95%CI = 1.582–2.698, *p* < 0.001) (Figure 2I).

### Comparison of Molecular Profile Mutations

To gain insight into the mutational mechanisms of HNSCC, we calculated the tumor mutation burden (TMB) as the number of somatic mutations for each patient. However, patients in the high- and low-risk score subgroups showed no significant difference in TMB (*p* = 0.07) (Figure 3A) or number of mutated genes (*p* = 0.067) (Figure 3B). Given a set of mutational signatures, we calculated the presence of somatic mutations to reveal signatures for these five genes (TP53, CDKN2A, SYNE1, PKHD1L1, and PEG3) and found nonsense mutations and missense mutations to be the most common mutations. In addition, the most frequent genetic lesion in HNSCC was in TP53, followed by CDKN2A, SYNE1, PKHD1L1, and PEG3 (Figure 3C).

### Clinical Signatures Between Different Risk Score Subgroups

**FIGURE 4:**
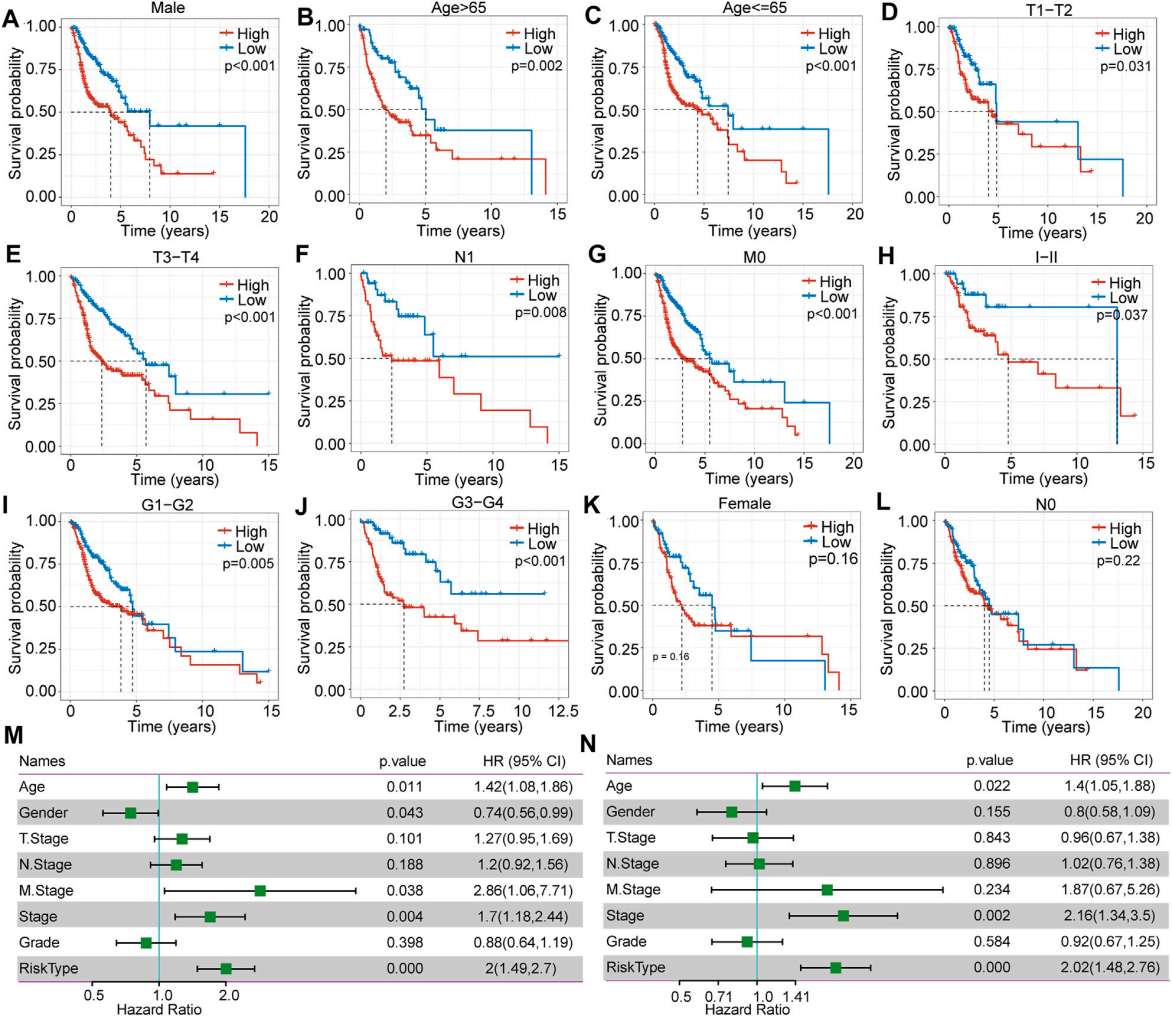
The difference in clinical signature-based prognosis between high- and low-risk score subgroups in TCGA-HNSC. **(A–L)** The overall survival comparison between the high- and low-risk score subgroups with regard to female, male, age >65, age ≤65, T1-T2, T3-T4, N0, N1, M0, MI-MII, G I-II, and G III-IV. **(M)** Correlations between clinical features and risk score through univariate regression analysis. **(N)** Correlations between clinical features and risk score through multivariate regression analysis.

In further investigating clinical signatures between the high- and low-risk score subgroups, we determined that there was no significant difference in T stage (*p* = 0.62), N stage (*p* = 0.84), M stage (*p* = 0.39), sex (*p* = 0.62), or age (*p* = 0.98) (Supplementary Figure 1A-F). In contrast, a significant difference in grade was observed (*p* = 0.00045) (Supplementary Figure 1G). In addition, we further analyzed overall survival as related to the clinical signature between these two subgroups. The Kaplan mire curve revealed a significant difference for male sex (*p* < 0.0001) **(** Figure 4A), age over 65 years (*p* = 0.0023) (Figure 4B), younger and equal to 65 years old (*p* = 0.00044) (Figure 4C), T1-T2 stags (*p* = 0.031), T3-T4 stages (*p* < 0.0001) (Figure 4E), N1 stage (*p* = 0.0078) (Figure 4F), M0 stage (*p* < 0.0001) (Figure 4G), M1-M2 stages (*p* = 0.037) (Figure 4H), grade 1–2 (*p* = 0.0053) (Figure 4I), and grade 3–4 (*p* = 0.00083) (Figure 4J). Overall survival analysis showed no significant difference between the high- and low-risk score subgroups for female sex e and N0 stage (Figure 4L).

### Clinical Signature Independent Validation

To assess the independence of the clinical signature as a prognostic factor, we applied multivariate analyses and the Cox proportional hazards regression model and determined that clinical characteristics such as age (*p* = 0.022, HR = 1.4 (1.05.1.88)), stage (*p* = 0.002, HR = 2.16 (1.34.3.5)) (Figure 4M and N), and risk score type were significantly associated with prognosis. Overall, the seven-gene signature model has good predictive value for HNSCC.

### Signaling Pathway Enrichment Analysis

**FIGURE 5:**
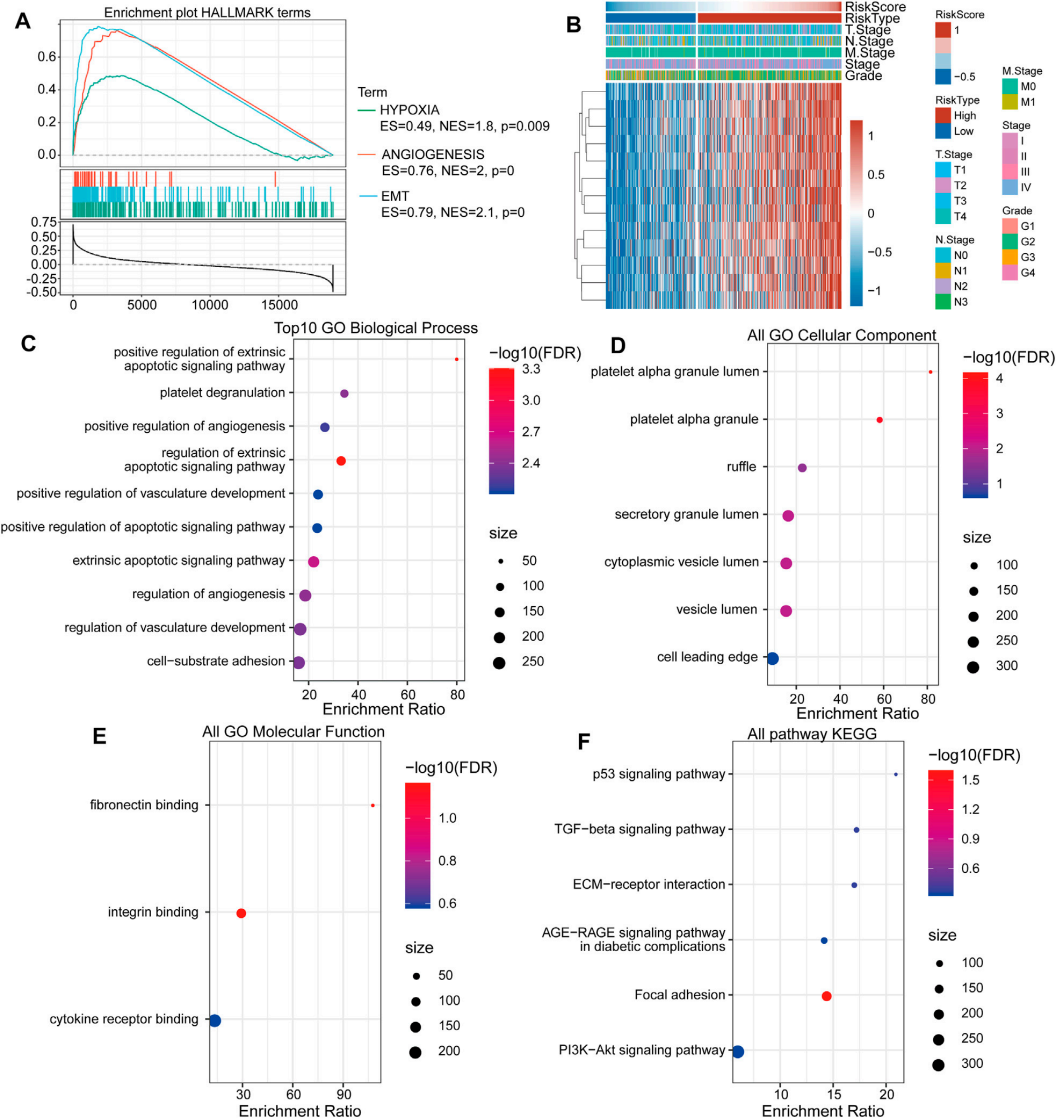
The TGF-β pathway gene-based high-risk score subgroup is related to tumor-associated biological signaling pathways. **(A)** The “HALLMARK” term enrichment plot between the high- and low-risk score subgroups. **(B)** The heat map shows the most correlating gene expression levels in the subgroups. **(C)** The enrichment ratio of the top ten gene ontologies through GO-BP analysis. **(D)** The enrichment ratio of the top ten gene ontologies through GO-CC analysis. **(E)** The enrichment ratio of the top ten gene ontologies through GO-MF analysis. **(F)** The enrichment ratio of the top ten gene ontologies through KEGG analysis.

When analyzing signaling pathway enrichment, we found that the high-risk score subgroup was enriched in hypoxia and EMT pathways (Figure 5A). To deeply explore prognosis-associated biological pathways, we obtained 13 positively correlating genes. The heatmap in Figure 5B illustrated that the expression level of these genes in the high- and low-risk score subgroups. GO classification includes biological process (GO-BP), cell component (GO-CC), and molecular function (GO-MF) categories. We identified 10 pathways as enriched in BP annotations (Figure 5C), seven in CC annotations (Figure 5D) and three in MF annotations (Figure 5E). KEGG analysis showed six biological signaling pathways to be involved in HNSCC regulation (Figure 5F).

### Immune Cell Infiltration and Inflammatory Features

**FIGURE 6:**
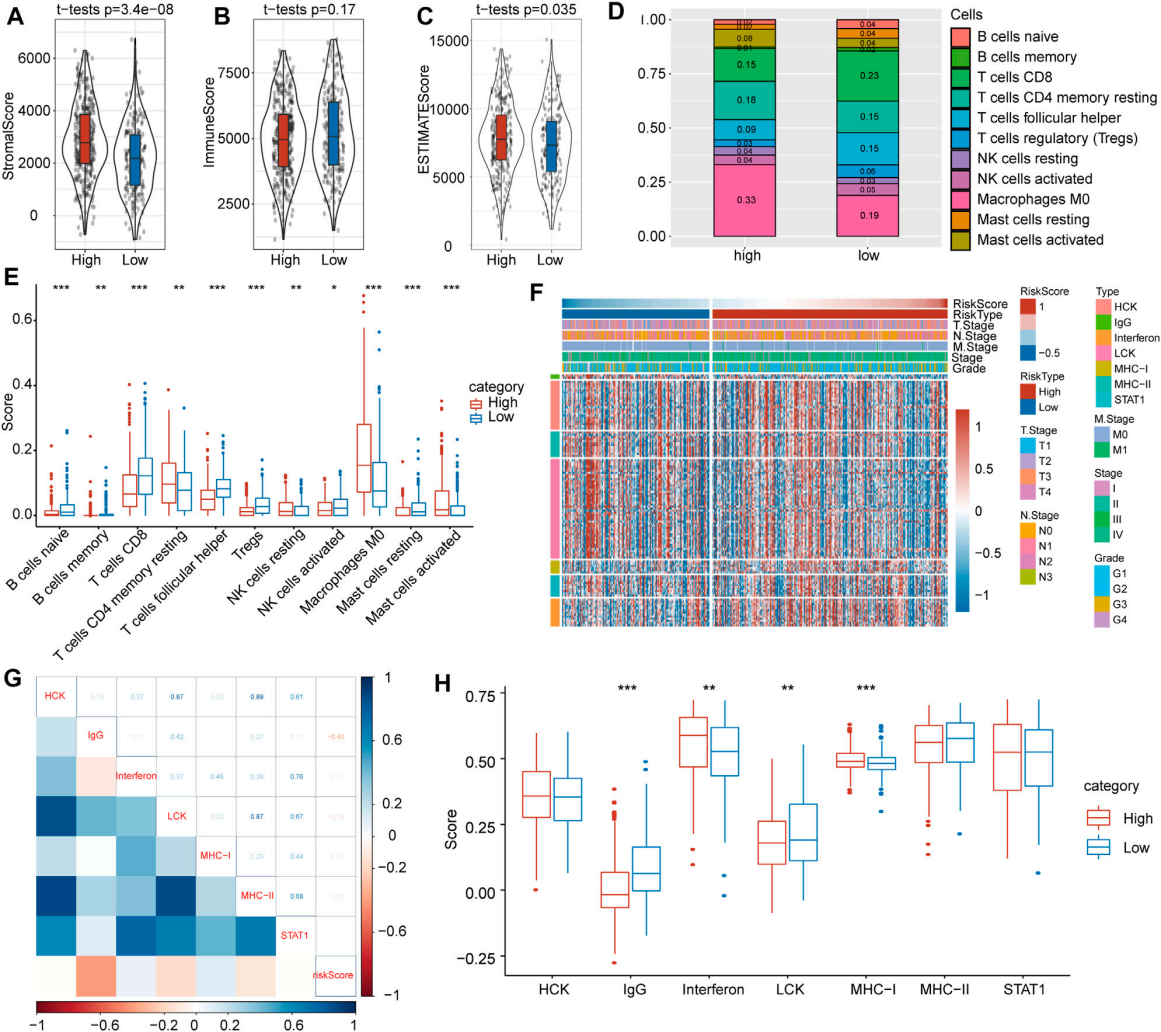
Immune-associated evaluations between high- and low-risk score subgroups. **(A)** The stromal score was greatly higher in the high-risk score subgroup. **(B)** There was no significant difference in immune score between the high- and low-risk subgroups. **(C)** The immune score was markedly higher in the high-risk score subgroup. **(D)** The immune cell distribution in the high- and low-risk subgroups. **(E)** Comparisons of involved immune cells between the two subgroups. **(F)** Heat map landscape of clinical feature distribution in the high- and low-risk score subgroups. **(G)** Correlations between ssGSEA scores of seven immune-associated genes. **(H)** Score comparisons of immune-associated genes between the high- and low-risk subgroups.

To investigate the relationship between risk score and immune cell infiltration and inflammatory characteristics in patients with HNSCC, we evaluated three immune microenvironment scores, namely, the stromal score, immune score, and ESTIMATE score, and found that the stromal score (Figure 6A) and ESTIMATE score (Figure 6B) were significantly higher in the high-risk group than in the low-risk group. Conversely, no significant difference was found for the immune score (Figure 6C). Moreover, immune cell infiltration evaluations indicated significant differences in the proportions of 11 types of immune cells in the subgroups (Figures 6D,E). Among them, the proportions of resting memory CD4^+^ T cells, resting NK cells, M0 macrophages and activated mast cells in the low-risk group were significantly lower than those in the high-risk group, whereas proportions of natural B cells, memory B cells, CD8^+^ T cells, follicular helper T cells, activated NK cells, regulatory T cells and resting mast cells were significantly higher in the low-risk group.

To probe inflammatory activity associated with risk scores, we examined relationships between seven metagene clusters, whereby differences represent different inflammatory and immune responses. The characteristics based on the TGF-β pathway were explored, and detailed information on these metagenes is provided in Figure 6F.

To verify the gene expression details observed, gene set variation analysis (GSVA) was utilized to calculate scores for the corresponding clusters of seven metagene clusters. Our results showed that the risk score correlated positively with IgG, LCK and MHC-II and with IgG, interferon and MHC-I (Figure 6G). At the same time, we compared differences of these seven scores in the high- and low-risk subgroups and found that IgG and LCK scores were significantly higher in the latter but that interferon and MHC-I scores were significantly higher in the former (Figure 6H).

### Difference in Clinical Effect Between High- and Low-Risk Score Subgroups After Immunotherapy and Chemical Therapy

**FIGURE 7:**
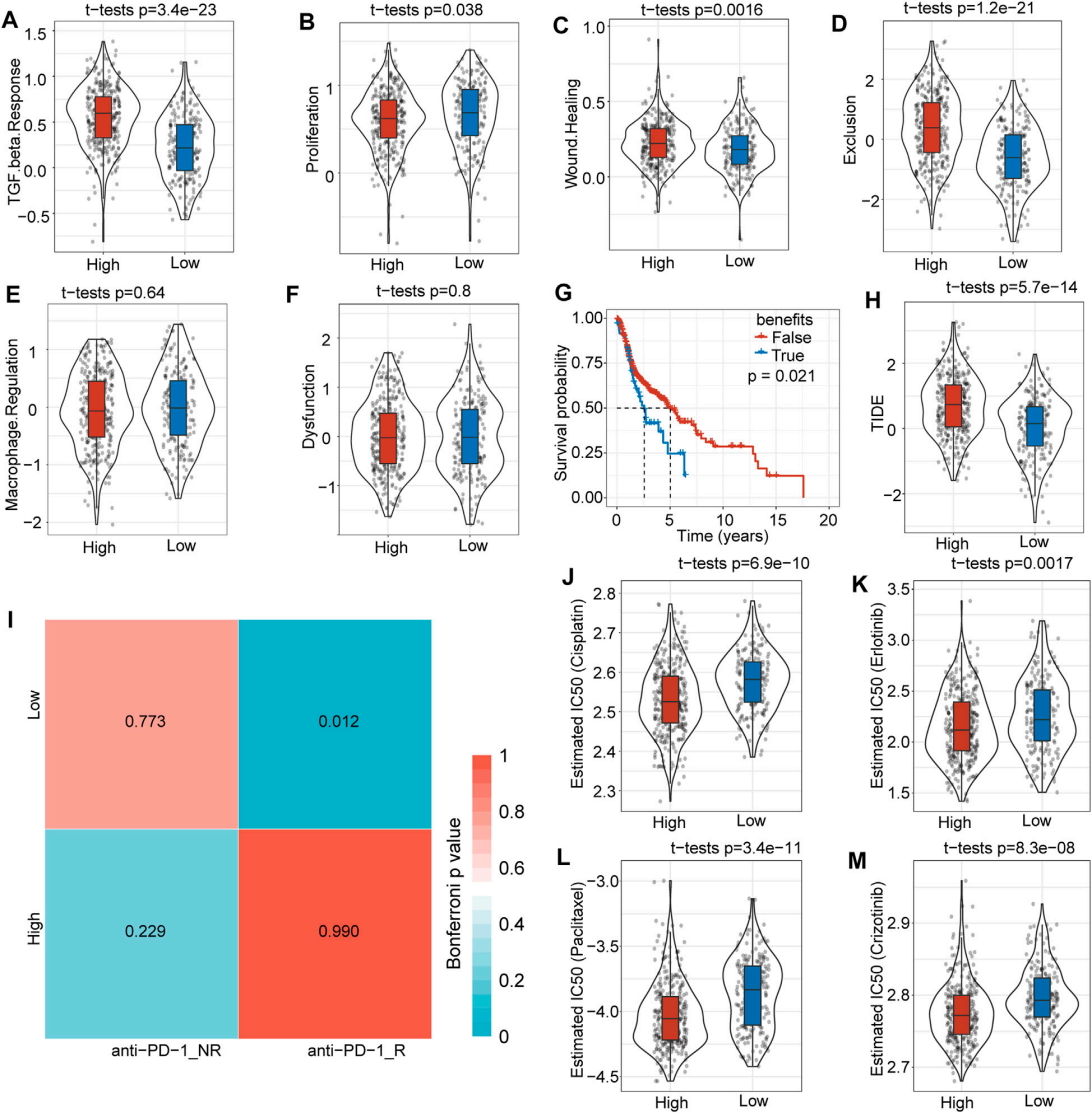
Differences in immunotherapy and clinical efficiency. **(A)** The TGF-β response was much higher in the high-risk score group than in the low-risk score group. **(B)** The proliferation score was slightly higher in the high-risk score subgroup. **(C)** There was no significant difference in macrophage regulation between the two subgroups. **(D)** The wound healing score was higher in the high-risk score subgroup. **(E)** Overall survival time prediction between true and false immunotherapies based on TIDE analysis tools. **(F)** The TIDE score was significantly higher in the high-risk score subgroup. **(G)** The dysfunction score was not significantly different between the two subgroups. **(H)** The exclusion score was much higher in the high-risk score subgroup. **(I)** Low-risk score subgroup patients might be more sensitive to anti-PD1 immunotherapy. **(J)** Box plots of the estimated IC50 for cisplatin showed that the high-risk score subgroup had a lower IC50 level. **(K)** Box plots of the estimated IC50 for erlotinib showed that the high-risk score subgroup had a lower IC50 level. **(L)** The box plots of estimated IC50 for paclitaxel showed that the high risk score subgroup had a lower IC50 level. **(M)** Box plots of the estimated IC50 for crizotinib showed that the high-risk score subgroup had a lower IC50 level.

To evaluate potential differences in clinical effects between immunotherapy and chemotherapy, we adopted TIDE software and found that the high-risk score subgroup had a markedly higher TGF-β response (*p* = 3.4e-23) (Figure 7A), proliferation score (*p* = 0.038) (Figure 7B), wound healing score (*p* = 0.016) (Figure 7C), and exclusion score (*p* = 1.2 e-21) (Figure 7D) compared with low-risk score subgroup. However, macrophage regulation (Figure 7E) and dysfunction (Figure 7F) showed no significant difference between the subgroups. The TIDE prediction therapy survival curve suggested that the FALSE group had a better prognosis (*p* = 0.021) (Figure 7G), and the TIDE score was much higher in the high-risk score group than in the low-risk score group (*p* = 5.7e-14) (Figure 7H).

We further analyzed differences in immunotherapy and chemotherapy among different subtypes of immune molecules. Subclass mapping was used to compare the similarity between the high-low risk subgroups in our TCGA-HNSC dataset and immunotherapy patients in the GSE78220 dataset: the lower the *p* value, the higher the similarity. For TCGA-HNSC, the low-risk score group was more sensitive to PD1 treatment (Figure 7I). This result was consistent with the TIDE results, indicating that low-risk group patients will benefit more from immunotherapy. The results also showed that the high-risk score subgroup was more sensitive to the traditional chemotherapy drugs cisplatin (*p* = 0.69e-10) (Figure 7J), erlotinib (*p* = 0.0017) (Figure 7K), paclitaxel (*p* = 3.4e-11) (Figure 7L) and crizotinib (*p* = 8.3e-08) (Figure 7M). Overall, this risk score model provides a novel basis for HNSCC patient treatment options.

## Discussion

TGF-β is involved in many biological functions in epithelial, endothelial, and neural tissues, in the immune system, and in wound repair (Massagué, 2012). TGF-β is a multifunctional cytokine, and its receptors play a crucial role in cancer initiation and progression through a range of activities in the regulation of cell proliferation, differentiation, apoptosis, and migration (Gencer et al., 2017). After activation of TGF-β signaling, TGF-β-associated ligands bind to corresponding receptors I and II (de la Cruz-Merino et al., 2009) and then transfer extracellular signals to nuclear components through canonical TGF-β pathways, such as the TGF-β/Smad pathway, and noncanonical TGF-β pathways, such as the p38/mitogen-activated protein kinase (MAPK) pathway, GTP pathway, PI3K/AKT pathway, and NF-κB pathway (Patil et al., 2011; Bataller et al., 2019; Chang and Pauklin, 2021; Choi et al., 2021; Hou et al., 2021). TGF-β has often been implicated in carcinogenesis, and studies have demonstrated that TGF-β has both oncogenic and tumor-suppressive functions in cancer regulation mechanisms (Yu and Feng, 2019; Belitškin et al., 2021). The antitumor ability of TGF-β functions occur through cytostatic and proapoptotic effects (Ahmadi et al., 2019). Inactivation of the antitumor function of TGF-β might lead to cancer initiation. Overexpression of TGF-β might have immunosuppressive effects on tumoral cells (Tsai et al., 2018), thus facilitating tumor progression in various cancers (Shao et al., 2018). Studies have reported that TGF-β pathway-associated genomic alterations account for approximately 40% of cancers (Korkut et al., 2018). TGF-β also play an important role in create an immunosuppressive tumor microenvironment. TGF-β signaling also reported play key role in mediating fibroblast phenotypic transformation through NOX4 in related to Human papillomavirus associated HNSCC patients (Wang et al., 2022). The TGF-β associated genes function as important tumor-microenvironment factors, and have been reported that activate the increased expression of the EMT transcription factor Slug in HNSCC (Ingruber et al., 2022).

In the present study, we demonstrated that in HNSCC, TGF-β-associated genes are related to a high TMB. Based on 54 TGF-β pathway-related genes, we constructed a 7-gene prognostic risk model, which exhibited stable robustness in internal and external validation sets. Furthermore, this model was to well predict HNSCC prognosis.

Immunotherapies can provide great benefit to patients who respond. Immunotherapeutics, such as immune checkpoint inhibitors, are considered to stimulate immune-mediated anticancer reactivity by interrupting the immune inhibitory pathway. Immunotherapies may result in long-term tumor regression, but the overall response rates are limited, especially for solid tumors (Baysal et al., 2021). HNSCC is an immunosuppressive disease, and immune checkpoint inhibitors are emerging as a promising therapy for patients with HNSCC. Studies have reported that recurrent/metastatic HNSCC has a better response to combination and single treatments, such as cetuximab/platinum/5-FU, pembrolizumab/platinum/5-FU or pembrolizumab alone. Nivolumab also shows better efficacy than other single agents (Wang H. et al., 2021; Hsieh et al., 2021), and cetuximab has an established role in HNSCC treatment (Hsieh et al., 2021).

In general, the discovery of predictive biomarkers and prognosis-related gene models may provide novel clues regarding presurgical and immunotherapy efficiency decision-making processes for individual patients.

In this study, we adopted GSEA to analyze pathways in high- and low-risk subgroups, among which tumor-related pathways were more enriched in the former, such as hypoxia and the EMT pathways. We calculated and functionally annotated genes associated with risk. Through TIDE and submap mapping analyses, we found that the low-risk group may be more suitable for immunotherapy and that the high-risk group is more sensitive to cisplatin, erlotinib, paclitaxel and crizotinib, as based on IC50 analysis.

In conclusion, our findings demonstrate that a TGF-β-associated gene-based prediction model has good efficiency for HNSCC clinical immunotherapy decision making and prognosis prediction.

## Data Availability

The datasets presented in this study can be found in online repositories. The names of the repository/repositories and accession number(s) can be found in the article.

## Supplementary Material

Supplementary Figure S1 Comparisons of risk scores among clinical features. **(A)** Comparison of risk scores among T stage-based subgroups. **(B)** Comparison of risk scores among N stage-based subgroups. **(C)** Comparison of risk scores among M stage-based subgroups. **(D)** Comparison of risk scores among clinical stage-based subgroups. **(E)** Comparison of risk scores among sex-based subgroups. **(F)** Comparison of risk scores among age-based subgroups. **(G)** Comparison of risk scores among grade-based subgroups.
