# Supplementary material for: TGF-β Signaling Pathway-Based Model to Predict the Subtype and Prognosis of Head and Neck Squamous Cell Carcinoma
Source: Front Genet. 2022 May 2;13:862860. doi: 10.3389/fgene.2022.862860 (PMC9108263; doi:10.3389/fgene.2022.862860)
Supplement: Supplementary file 1 [file Table1.docx]

Table S1. The information of primers sequences for qRT-PCR assay.

| Primer name | Sequence (5'-3') |
| --- | --- |
| TRIM33-F | TGGAGGAGGAGGAGGAGGAAGG |
| TRIM33-R | GCAGGCAGAAGGAGTGAAGACAG |
| ID2-F | CAGTCCCGTGAGGTCCGTTAGG |
| ID2-R | CTCTGGTGATGCAGGCTGACAATAG |
| THBS1-F | ATCAGGCAGACACAGACAACAATGG |
| THBS1-R | CATCATCGTGGTCACAGGCATCTC |
| SLC20A1-F | GTCCAGTTCAGTCAAGCCGTCAG |
| SLC20A1-R | TTTCTTCGCCCTTCTGTTCACCTTC |
| SERPINE1 -F | GTCTTTGGTGAAGGGTCTGCTGTG |
| SERPINE1 -R | GCGTCTGTGGTGCTGATCTCATC |
| NOG-F | CGAACACCCAGACCCTATCTTTGAC |
| NOG-R | TGTAACTTCCTCCGCAGCTTCTTG |
| BCAR3-F | CAGTGGGACAAGGGCGAGTTTG |
| BCAR3-R | CCGTGAGGCAAGGTAATGAGTTCC |
