# Supplementary figures and images for: TGF-β Signaling Pathway-Based Model to Predict the Subtype and Prognosis of Head and Neck Squamous Cell Carcinoma
Source: Front Genet. 2022 May 2;13:862860. doi: 10.3389/fgene.2022.862860 (PMC9108263; doi:10.3389/fgene.2022.862860)

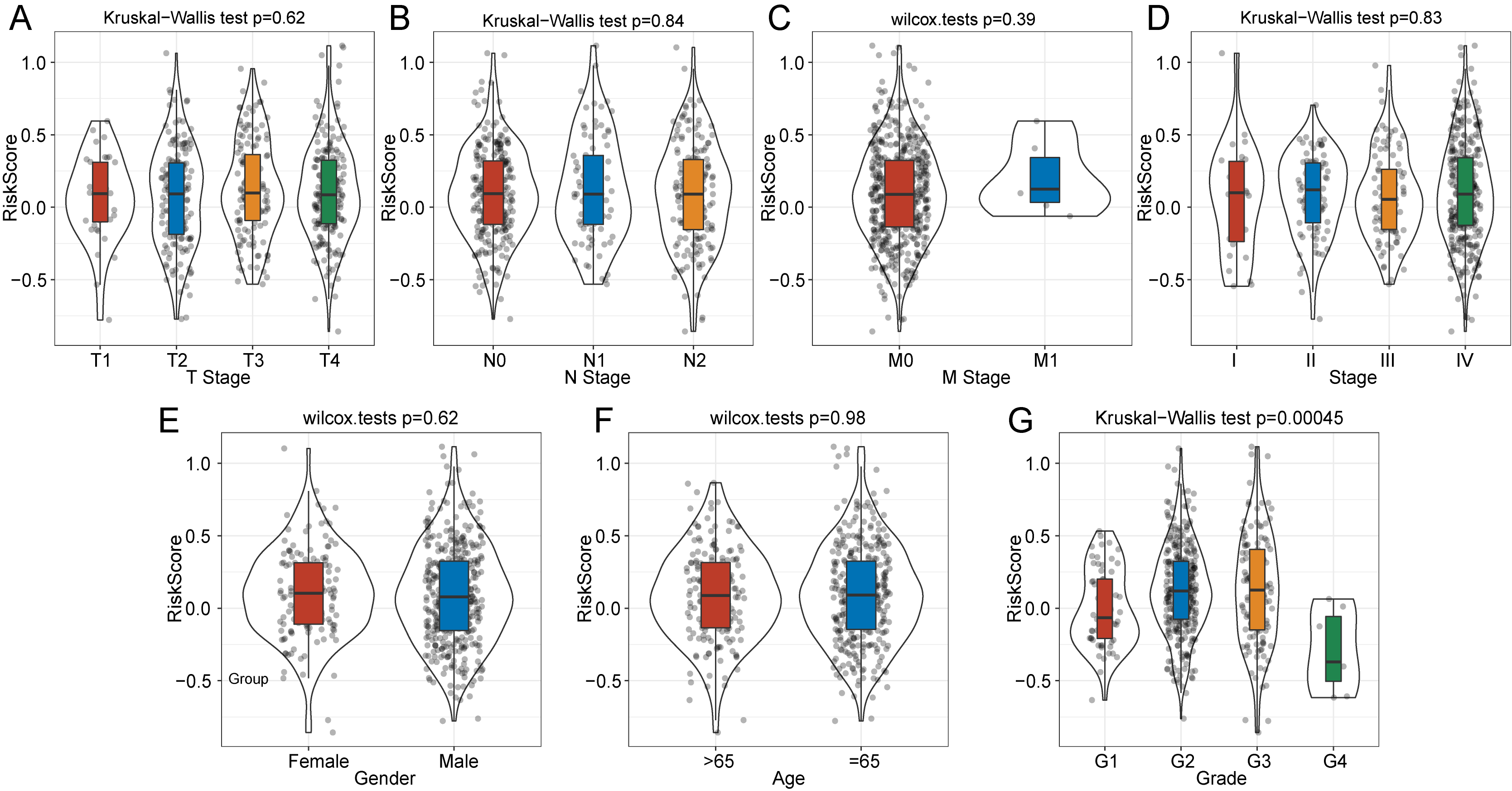

Supplement: Supplementary file 2 [file Image1.TIF]
